# Earth’s Subdecadal Angular Momentum Balance from Deformation and Rotation Data

**DOI:** 10.1038/s41598-018-32043-8

**Published:** 2018-09-13

**Authors:** Andrew Watkins, Yuning Fu, Richard Gross

**Affiliations:** 1 Department of Geology, School of Earth, Environment and Society, Bowling Green State University, Bowling Green, OH 43403 USA; 2 Jet Propulsion Laboratory, California Institute of Technology, Pasadena, CA 91109 USA

## Abstract

Length-of-Day (LOD) measurements represent variations in the angular momentum of the solid Earth (crust and mantle). There is a known ~6-year LOD signal suspected to be due to core-mantle coupling. If it is, then the core flow associated with the 6-year LOD signal may also deform the mantle, causing a 6-year signal in the deformation of the Earth’s surface. Stacking of Global Positioning System (GPS) data is found to contain a ~6-year radial deformation signal. We inverted the deformation signal for the outer core’s flow and equivalent angular momentum changes, finding good agreement with the LOD signal in some cases. These results support the idea of subdecadal core-mantle coupling, but are not robust. Interpretation of the results must also take into account methodological limitations. Gravitational field changes resulting from solid Earth deformation were also computed and found to be smaller than the errors in the currently available data.

## Introduction

The Length-of-Day (LOD) exhibits subtle fluctuations on a variety of timescales. Conservation of angular momentum applied to the solid Earth (crust and mantle) requires either mass redistribution or some external torque to explain these LOD fluctuations^1^. Previous investigations have established the outer core as one important source of torque on the solid Earth. In these previous investigations, geomagnetic field variations were inverted for the outer core’s flow and angular momentum L_OC_. Researchers found that a torque coupling the ΔL_OC_ to the solid Earth would cause LOD changes that agree well with measured ΔLOD on decadal timescales^1–3^.

There is a ~6-year LOD signal that remains after removing the effects of the oceans and atmosphere^1^. Both the fluid outer core^4^ and the solid inner core^5^ have been suggested as causes for the signal. This study aimed to 1) Test the idea that the ~6-year LOD signal is due to angular momentum exchange between the solid Earth and outer core, and 2) Demonstrate the viability of a novel approach to investigating the core’s rotation: the inversion of crustal deformation data.

**Figure 1:**
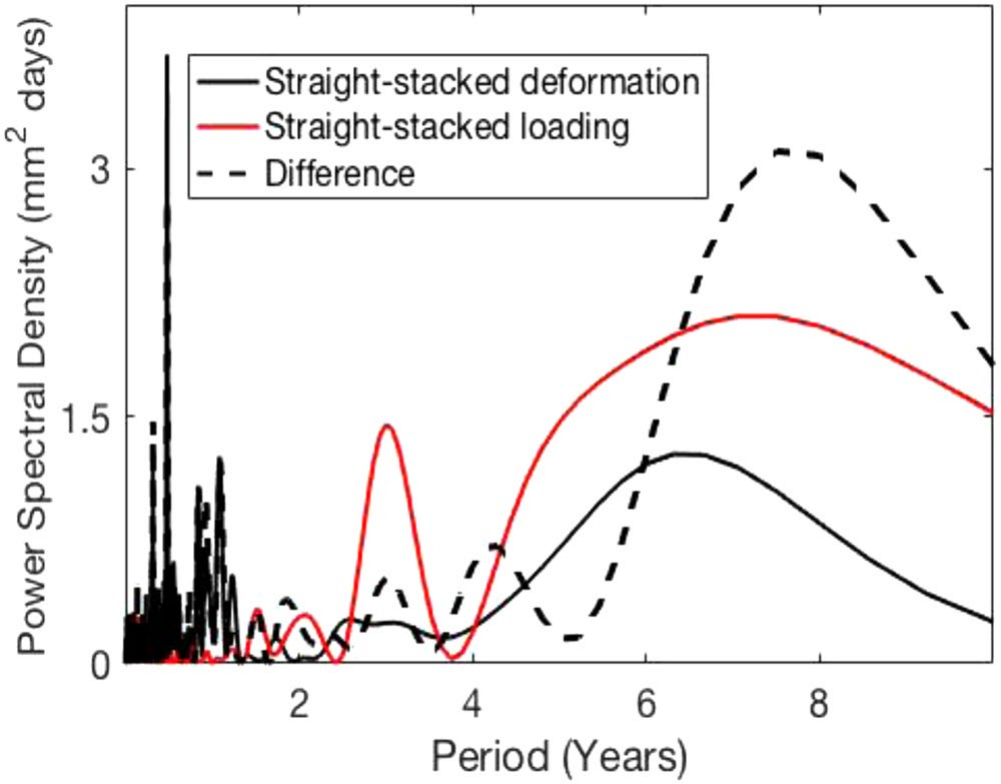
The spectrum of the straight-stack (arithmetic mean) of 523 GPS radial time series (black curve) contains a peak at ~6 years. Loading data (red curve) is modeled non-tidal atmospheric, non-tidal oceanic, and hydrological contributions. See the Supplementary Notes for additional details.

The Jet Propulsion Laboratory (JPL) produces position solutions for a globally distributed network of Global Positioning System (GPS) stations (https://sideshow.jpl.nasa.gov/post/series.html). We analyzed the radial component of JPL’s residual time series during the time period 1 January 2002–2014. We analyzed the spectrum of 523 stacked GPS radial time series, and found there is a global ~6-year deformation signal (Fig. 1). Modeled surface loading data^6^ (red curve in Fig. 1) from the German Research Centre for Geosciences (https://www.gfz-potsdam.de/en/esmdata/loading/) was bilinearly interpolated to the station locations, and does not account for the ~6-year deformation signal.

Surface loading does not account for the ~6-year deformation signal, so the cause may be located within Earth’s interior. Fang *et al*.^7^ detailed how pressure anomalies below the core-mantle boundary (CMB) deform the solid Earth. Using this mathematical framework, we modeled the CMB pressure fluctuations that would explain the deformation. Geostrophic flow solutions were computed from the pressure field, and the flow at depth within the core was assumed to be organized into nested cylindrical annuli. Equivalent ΔL_OC_ was derived from the flow field.

The ~6-year LOD signal was isolated and used to derive a time series of equivalent solid Earth angular momentum changes ΔL_MC_. Angular momentum conservation of a coupled outer core-solid Earth system gives a prediction: ${{\rm{\Delta }}L}_{{\rm{OC}}}=-\,{{\rm{\Delta }}L}_{{\rm{MC}}}$. Results were compared with this expectation, and gravitational field changes resulting from solid Earth deformation were computed.

## Methods

### Solid Earth Angular Momentum

We used JPL’s daily COMB2015 Noon dataset^8^ (https://keof.jpl.nasa.gov) to isolate the LOD signal. We first corrected for the effects of the ocean and atmosphere:

1 $${{\rm{\Delta }}\mathrm{LOD}}_{{\rm{corrected}}}={\rm{\Delta }}\mathrm{LOD}-{{\rm{\Delta }}\mathrm{LOD}}_{{\rm{oceans}}}-{{\rm{\Delta }}\mathrm{LOD}}_{{\rm{atmosphere}}}$$

The ΔLOD_oceans_ term is provided directly by the International Earth Rotation and Reference Systems Service’s (IERS) Special Bureau for the Oceans (https://euler.jpl.nasa.gov/sbo/sbo_home.html). This study used the (daily) ECCO_kf080h.chi dataset for 2 January 1993 and onward, and linearly interpolated the ECCO_50yr.chi dataset (10-day sampling interval) to daily for prior dates. The ${\rm{\Delta }}$LOD_atmosphere_ accounts for atmospheric angular momentum (AAM) variations and was computed as^9^:

2 $${{\rm{\Delta }}\mathrm{LOD}}_{{\rm{atmosphere}}}=\,\frac{(86400\,s)}{{{\rm{C}}}_{{\rm{mc}}}{\bar{{\rm{\omega }}}}_{{\rm{E}}}}{\rm{\Delta }}\mathrm{AAM}$$

where C_mc_ is the solid Earth’s axial moment of inertia and ${\bar{{\rm{\omega }}}}_{{\rm{E}}}$ is Earth’s mean rotation rate. Raw AAM data is from the Reanalysis Project^10–13^ of the National Center for Environmental Protection (NCEP) and the National Center for Atmospheric Research (NCAR) (http://www.aer.com/science-research/earth/earth-mass-and-rotation/special-bureau-atmosphere). The ${{\rm{\Delta }}\mathrm{AAM}}_{{\rm{mass}}}$ term with the inverted-barometer correction was used. Raw data was reported in six-hour intervals. Five consecutive values were added with weights: 1/8, 1/4, 1/4, 1/4, and 1/8 (respectively), centering the daily average at noon.

A 3^rd^-order Savitzky-Golay filter with a 1095-day frame was then applied to smooth the dataset. The power spectral density of the smoothed LOD then displayed a peak at 5.85 years. This desired signal was extracted via a curve fit of the form ${\rm{\lambda }}\,\cos (2{\rm{\pi }}t/{\rm{T}}+{\rm{\phi }})$, where ${\rm{T}}=5.85\,\mathrm{years}=2,136.71\,\mathrm{days}$. An equivalent ${{\rm{\Delta }}L}_{{\rm{MC}}}$ signal was formed following equation (2) by replacing ΔLOD_atmosphere_ with the fitted signal and ΔAAM with −ΔL_MC_.

### Deformation Signal

The GPS stations used in this study were those with no more than 30% of days missing data and no gaps larger than 365-days during the 12-year period under consideration. Linear interpolation filled these gaps, and interpolated points were assigned an error estimate of 1 cm.

Monthly (31-day) averages (weighted by inverse-variance) were then taken. A 12-sample moving average was then used to smooth the data using the previous six data points, the data point in question, and the following five data points. Accordingly, the time stamp for the sample is moved 0.5 samples (15.5 days) backwards. The endpoints where the 12-sample window could not be defined consistently were discarded.

A curve of the form ${\rm{\lambda }}\,\cos (2{\rm{\pi }}t/{\rm{T}}+{\rm{\phi }})$ was then fit to the time series, where ${\rm{T}}=6\,\mathrm{years}=2,\,191.5\,\mathrm{days}$. This curve was taken as the desired signal.

### CMB Pressure Anomalies

The radial deformation Δr from CMB pressure anomalies p was given by Fang *et al*.^7^. As in Fang *et al*. (1996), we considered the core-mantle boundary and crust to be spherical. We considered a finite number of pressure and deformation grid cells on the sphere, and used a discretized form of the equation:

3 $${{\rm{\Delta }}r}_{{\rm{i}}}=\,\frac{3}{4{{\rm{\pi }}g\bar{{\rm{\rho }}}}_{{\rm{E}}}}[{{\rm{p}}}_{1}{{\rm{dQ}}}_{1}{\sum }_{{\rm{n}}=1}^{10}{{\rm{h}}}_{{\rm{n}}}{{\rm{P}}}_{{\rm{n}}}(\cos \,{{\rm{\alpha }}}_{{\rm{i}}2})+\ldots +{{\rm{p}}}_{{\rm{s}}}{{\rm{dQ}}}_{{\rm{s}}}{\sum }_{{\rm{n}}=1}^{10}{{\rm{h}}}_{{\rm{n}}}{{\rm{P}}}_{{\rm{n}}}(\cos \,{{\rm{\alpha }}}_{{\rm{in}}})]$$

where g is the gravitational acceleration at Earth’s surface, ${\bar{{\rm{\rho }}}}_{{\rm{E}}}$ is the average density of Earth, dQ_i_ is the area of the i^th^ grid cell on a unit sphere, h_n_ is the Love number h of degree n (the data source is given in the Supplementary Materials), P_n_ is the Legendre polynomial of degree n, and α_ij_ is the arc length between the i^th^ and j^th^ grid cell centers on a unit sphere. A set of m deformation samples gives rise to a linear system:

4 $${{\rm{\Delta }}r}_{{\rm{mx}}1}={{\rm{C}}}_{{\rm{mxs}}}{{\rm{p}}}_{{\rm{sx}}1}$$

where

5 $${{\rm{C}}}_{{\rm{ij}}}=\frac{3{{\rm{dQ}}}_{{\rm{j}}}}{4{{\rm{\pi }}g\bar{{\rm{\rho }}}}_{{\rm{E}}}}{\sum }_{{\rm{n}}=1}^{10}{{\rm{h}}}_{{\rm{n}}}{{\rm{P}}}_{{\rm{n}}}(\cos \,{{\rm{\alpha }}}_{{\rm{ij}}})$$

The Δr_i_ were populated by taking the inverse-variance-weighted average of the deformation signal for all GPS stations lying within the boundaries of the i^th^ grid cell. The CMB pressure field was then modeled by inverting the linear system: ${{\rm{p}}}_{{\rm{est}}}={{\rm{C}}}^{-1}{\rm{\Delta }}r$.

We compensated for the sparse spatial distribution of GPS stations by inverting six distinct linear systems, each with large grid cells (see Supplementary Table S1 for details). The layout for each inversion was staggered from the other inversion layouts. There was overlap between the grids of the different inversions, but by staggering the layout, each cell sampled a unique set of GPS stations (except for pole samples), and each linear system was fully determined. For each system, the grid layout on Earth’s surface was the same as that on the CMB.

The pressure samples from all six inversions were then combined onto the same sphere. Three of the inversions had samples at the North and South poles. In these cases, an inverse-variance-weighted average was used.

### Geostrophic Flow

Flow solutions were derived from the pressure field based on the assumption of tangentially geostrophic flow (where Coriolis and pressure gradient forces dominate) in the outermost core. The equation governing this assumption is^14^:

6 $${\bf{u}}=\frac{{\bf{n}}\times {\nabla }_{{\rm{H}}}{\rm{p}}}{2{{\rm{\rho }}}_{{\rm{OC}}}{\bar{{\rm{\omega }}}}_{{\rm{E}}}\,\cos \,{\rm{\theta }}}$$

where **u** is the flow vector, **n** is the unit normal vector, ${\nabla }_{{\rm{H}}}$ is the horizontal gradient operator, ${{\rm{\rho }}}_{{\rm{OC}}}$ is the outer core’s density, and ${\rm{\theta }}$ is the colatitude. The geostrophic assumption does not apply on the equator (note the cos ${\rm{\theta }}$ factor in the denominator). As will be discussed in the following section, equatorial samples will be used to represent a broader region around the equator extending 15° to the North and South. Thus, the zonal component of the flow in the region around the equator is approximated by computing flow samples at the equator with ${\rm{\theta }}$ set to 82.5°.

We used a discrete substitute for the horizontal gradient operator ${\nabla }_{{\rm{H}}}$ allowing a computation of the flow directly from equation (6). The details of the computation of ${\nabla }_{{\rm{H}}}{\rm{p}}$ are included in the Supplementary Notes.

The flow at depth within the outer core was solved by considering a finite set of nested geostrophic cylinders^15^. Three cylindrical annuli were defined by considering their intersection with the CMB. Each annulus intersects the CMB at two latitudes (a polar edge and an equatorial edge) in both the northern and southern hemispheres (except for one equatorial annulus, which has just one edge in each hemisphere). The polar edge of the first annulus was taken to be the boundary latitude of the cylinder tangent to the inner core (which is $\arccos ({{\rm{r}}}_{{\rm{ICB}}}/{{\rm{r}}}_{{\rm{CMB}}})$, where ${{\rm{r}}}_{{\rm{ICB}}}$ is the radius of the inner core and ${{\rm{r}}}_{{\rm{CMB}}}$ is the CMB radius). The remaining edges were placed halfway between latitude bands of flow samples.

Each flow sample rests on the surface of one annuli. An angular velocity vector ω was associated with each flow vector **u** according to:

7 $${\rm{\omega }}=\frac{|{\bf{u}}|}{|{\bf{r}}|}\frac{{\bf{r}}\times {\bf{u}}}{|{\bf{r}}\times {\bf{u}}|}$$

where **r** is the position vector of the flow sample **u**. For each annulus, an inverse-variance-weighted average of the z-components ${\bar{{\rm{\omega }}}}_{{\rm{z}}}$ was taken, and the entire annulus was assumed to be moving with this angular velocity.

### Outer Core Angular Momentum

**Figure 2:**
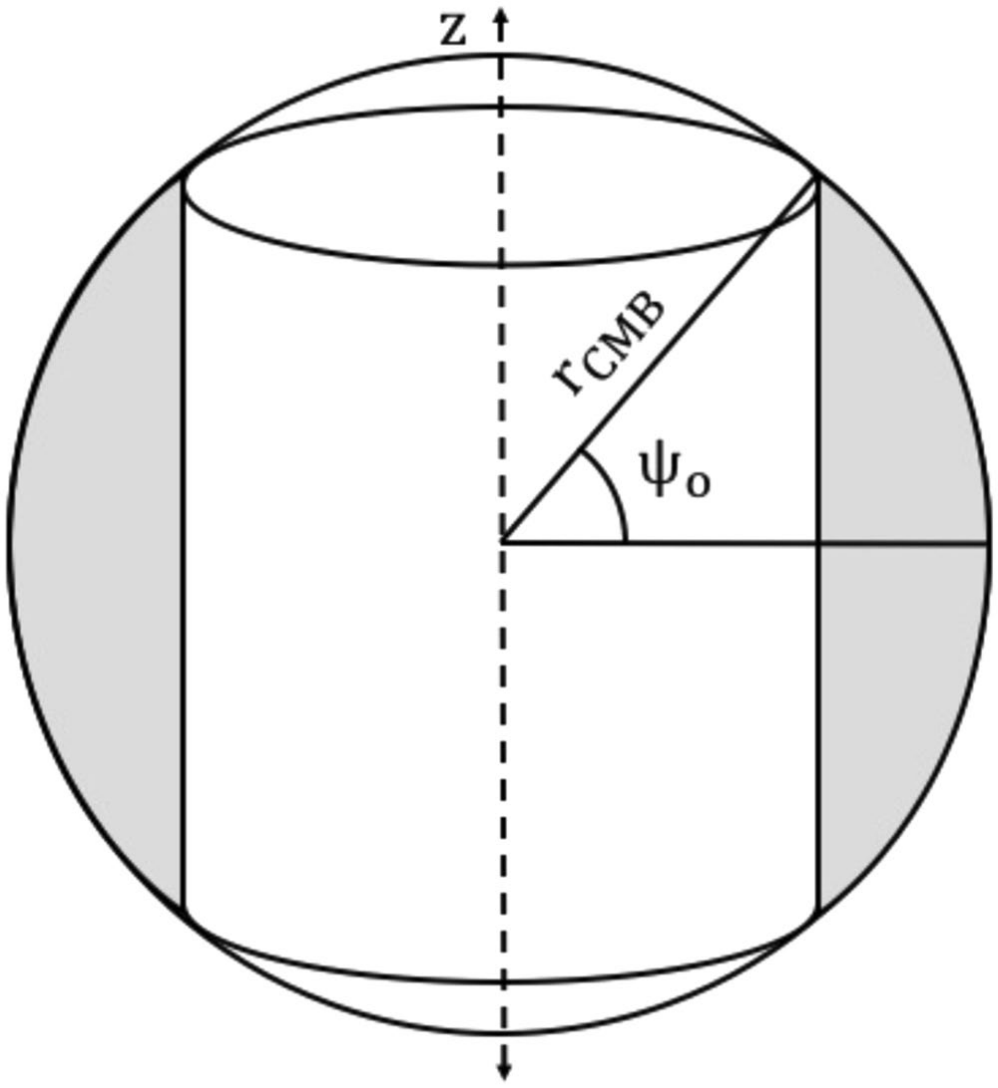
The axial moments of inertia are defined for cylindrical annuli (grey shaded region) that lie outside a single cylinder.

Consider an annulus of outer core fluid lying outside a single cylinder that intersects the CMB at latitudes of $\pm {{\rm{\psi }}}_{{\rm{o}}}$ (Fig. 2). The axial moment of inertia I_z_ of this annulus is (see the Supplementary Notes for proof):

8 $${{\rm{I}}}_{{\rm{z}}}({{\rm{\psi }}}_{{\rm{o}}})=\frac{4{{\rm{\pi }}{\rm{\rho }}}_{{\rm{OC}}}{{\rm{r}}}_{{\rm{CMB}}}^{5}}{5}\,\sin \,{{\rm{\psi }}}_{{\rm{o}}}(1-\frac{{\sin }^{2}{{\rm{\psi }}}_{{\rm{o}}}}{3}-{\cos }^{4}{{\rm{\psi }}}_{{\rm{o}}})$$

The axial moment of inertia for each annulus was formed by evaluating equation (8) at the equatorial edge, and subtracting that value from equation (8) evaluated at the polar edge. For the equatorial annulus, only one evaluation was necessary.

Summing ${{\rm{L}}}_{{\rm{z}}}={{\rm{I}}}_{{\rm{z}}}{\bar{{\rm{\omega }}}}_{{\rm{z}}}$ over all the annuli gave the ΔL_oc_. The mean was then removed from the ΔL_oc_ time series.

### Gravitational Field Changes

The solid Earth deformation from CMB pressure anomalies causes gravitational potential changes ΔU at Earth’s surface, detailed by Fang *et al*.^7^. We discretized these equations in a manner similar to equations (3–5), using a grid based on the combined set of pressure samples to forward-model the ΔU with a single linear system:

9 $${{\rm{\Delta }}U}_{{\rm{mx}}1}={{\rm{C}}}_{2,{\rm{mxs}}}{{\rm{p}}}_{{\rm{sx}}1}$$

where

10 $${{\rm{C}}}_{2,{\rm{ij}}}=\frac{3{{\rm{dQ}}}_{{\rm{j}}}}{4{{\rm{\pi }}\bar{{\rm{\rho }}}}_{{\rm{E}}}}{\sum }_{{\rm{n}}=1}^{10}{{\rm{k}}}_{{\rm{n}}}{{\rm{P}}}_{{\rm{n}}}(\cos \,{{\rm{\alpha }}}_{{\rm{ij}}})$$

where k_n_ is the Love number k of degree n. We express the gravity changes in terms of the normalized C_20_ coefficient, which describes Earth’s oblateness^16^. Equivalent oblateness changes were computed as (see the Supplementary Notes for proof):

11 $${{\rm{C}}}_{20}=\frac{{{\rm{r}}}_{{\rm{E}}}\sqrt{5}}{8{{\rm{GM}}}_{{\rm{E}}}{\rm{\pi }}}{\sum }_{{\rm{i}}}{{\rm{U}}}_{{\rm{i}}}({\varphi }_{{\rm{i}}1}-{\varphi }_{{\rm{i}}2})({\cos }^{3}{{\rm{\theta }}}_{{\rm{i}}2}-\,\cos \,{{\rm{\theta }}}_{{\rm{i}}2}-{\cos }^{3}{{\rm{\theta }}}_{{\rm{i}}1}+\,\cos \,{{\rm{\theta }}}_{{\rm{i}}1})\,$$

where r_E_ is Earth’s radius, G is the gravitational constant, M_E_ is Earth’s mass, and the coordinates with i1 and i2 subscripts are the lower and upper boundaries of the i^th^ grid cell, respectively. Values for these and other physical parameters used in this study are presented in Supplementary Table S2.

### Error Estimation

We used a general formula^17^ describing the standard error estimate ${{\rm{\sigma }}}_{{\rm{f}}}$ of a function f of a set of variables ${{\rm{\beta }}}_{{\rm{i}}}$:

12 $${{\rm{\sigma }}}_{{\rm{f}}}=\sqrt{{{\rm{g}}}^{{\rm{T}}}{\rm{Vg}}}$$

where g is a column vector whose i^th^ element is $\partial {\rm{f}}/\partial {{\rm{\beta }}}_{{\rm{i}}}$, and V is the (sample) covariance matrix among the β_i_. For vector quantities, error propagated through the magnitude only, and the cross product in equation (6) was considered equivalent to scalar multiplication when computing the g_i_. Error estimates for the deformation signal considered the λ and ${\rm{\phi }}$ as uncorrelated fit parameters. The errors ${{\rm{\sigma }}}_{{\rm{\lambda }}}$ and ${{\rm{\sigma }}}_{{\rm{\phi }}}$ were taken to be the half-width of the 68% confidence interval for the appropriate parameter.

### Alternate Inversions

In order to test the robustness of the results, we considered two 12-year time periods: 1 January 2002–2014 (the set used for Fig. 1), referred to as Inversion A, and 1 January 2004–2016, referred to as Inversion B.

## Results

**Figure 3:**
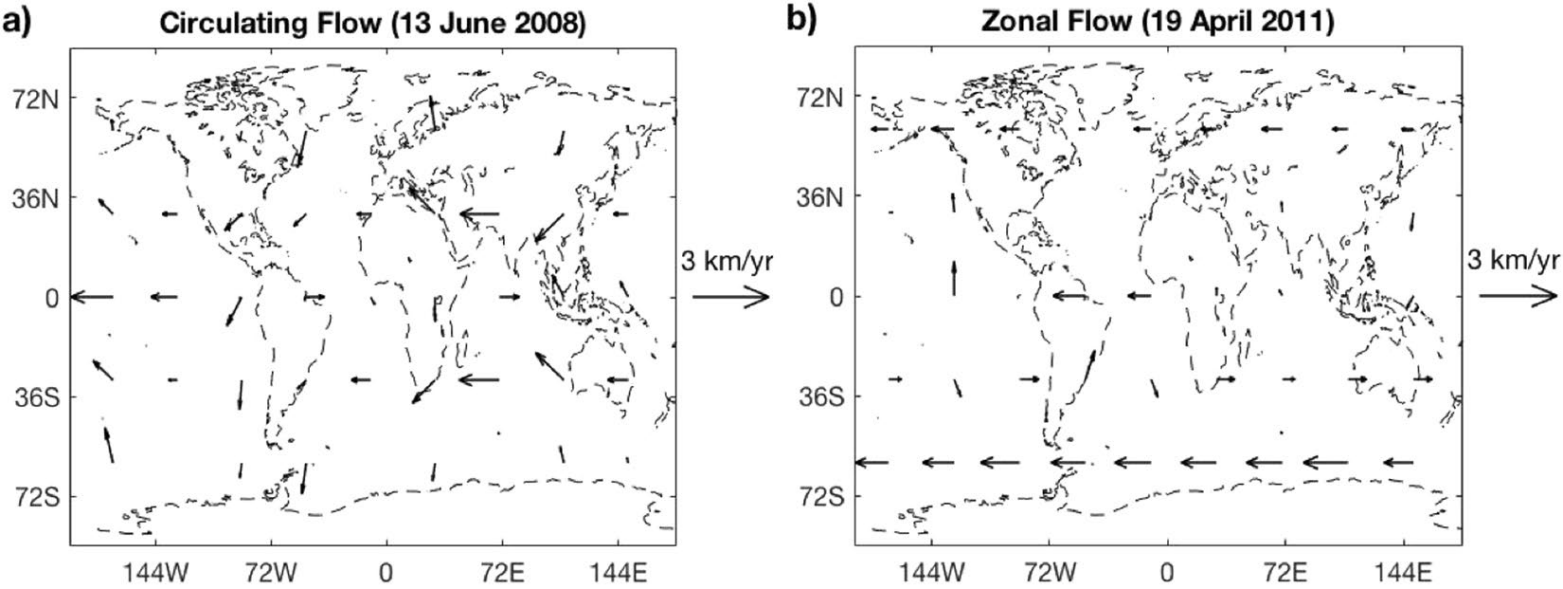
Geostrophic outer core flow solutions exhibit **a**) Circulating (taken from Inversion **A)**, **b**) Zonal (taken from Inversion **B**) features at latitudes of ±60°. Flow vectors at the equator have been scaled down to 1/2 of their original length for display, and should not be interpreted as representing the flow exactly at the equator. Instead, they approximate the zonal flow component in a broader equatorial region.

Inversion A had a mean deformation signal amplitude of 0.99 mm and a median of 0.84 mm, while Inversion B had a mean signal amplitude of 0.90 mm and a median of 0.76 mm. The estimated pressure variations were on the order of 10^2^ Pa, and the flow vectors on the order of 1km/yr. The flow solutions are generally westward and display a noticeable degree of symmetry about the equator. The flows can be broadly categorized as (1) Zonal, (2) Circulating, and (3) Chaotic. The former two are more common, and examples are shown in Fig. 3. The evolution over time of the flow solutions are shown in Supplementary Figures S2 and S3.

**Figure 4:**
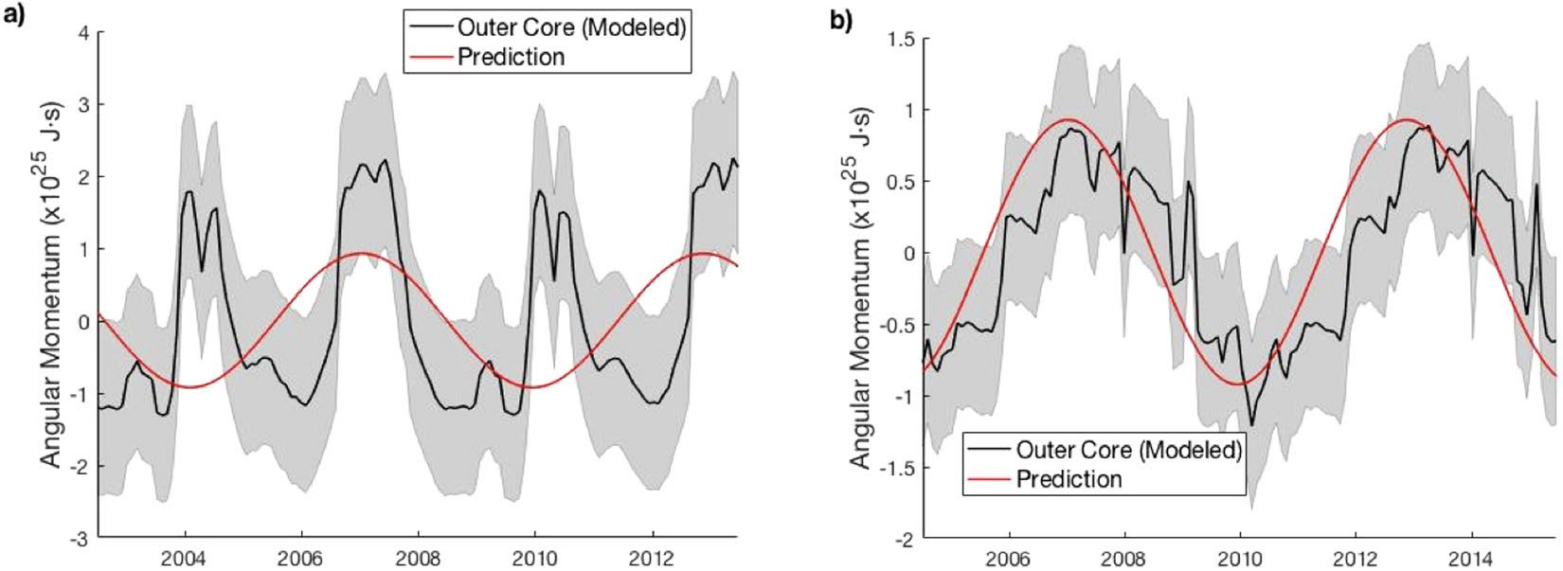
Outer core angular momentum solutions (**a**) from Inversion A and (**b**) from Inversion B. The latter agrees in phase with the prediction −ΔL_MC_ (correlation coefficient = 0.79). Grey shading represents standard 1-σ error estimates.

The fitted LOD signal at 5.85 years had an amplitude of 0.15 ms, corresponding to a ΔL_MC_ signal about 10^25 ^J s in amplitude. The computed ΔL_oc_ are on the same order of magnitude as the ΔL_MC_ signal. The ΔL_oc_ from Inversion A shows little phase relationship with the ΔL_MC_, while the ΔL_OC_ from Inversion B is opposite the ΔL_MC_ in phase (Fig. 4).

**Figure 5:**
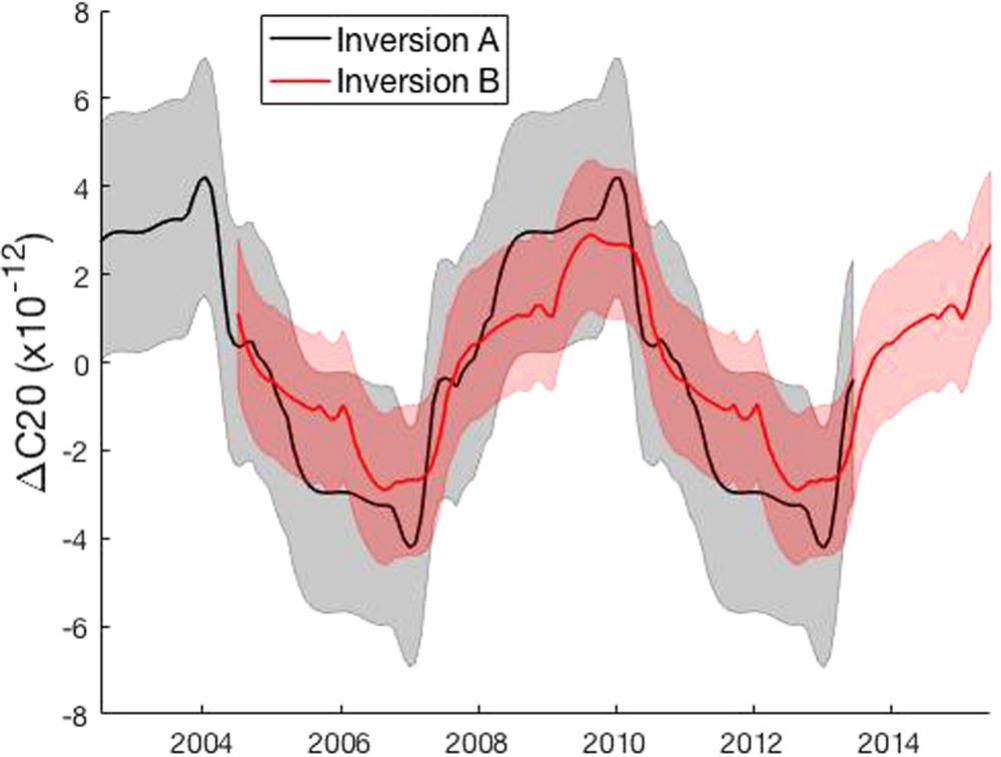
Computed Earth oblateness changes show a ~6-year signal and are robust in magnitude and phase. Standard 1-σ error estimates are shown in grey shading for Inversion A and in red shading for Inversion B.

The computed interannual Earth oblateness (C_20_) signal is on the order of 10^−12^ (Fig. 5), about one order of magnitude smaller than the standard errors in the currently available data^18^.

## Discussion and Conclusion

The LOD signal isolated in this study (see Supplementary Fig. S1) is about 25% larger than those from previous investigations, and is in general phase agreement since 2000^4,19,20^. The symmetry of the flow solutions about the equator is consistent with Taylor’s constraint. The westward nature of the solutions suggests they may be associated with the westward drift of the magnetic field. The westward drift undergoes decadal fluctuations that have been inverted for the core’s angular momentum, which agree with expectations from decadal LOD signals^21^. The circulating flows resemble circulations in some magnetic field inversions, which have been interpreted as evidence of columnar flow^22^. One ensemble inversion of geomagnetic field observations has examined the subdecadal timescale and found evidence that the outer core is the cause of the ~6-year LOD signal^23^.

The ΔL_OC_ from Inversion B (Fig. 4b) supports the idea that the ~6-year LOD signal is due to angular momentum exchange between the solid Earth and outer core. However, this result is not robust; The ΔL_OC_ from Inversion A (Fig. 4a) does not agree in phase with the prediction.

The two inversions have a significant overlap in time (1 Jan 2004–2014), during which the ΔL_OC_ solutions disagree. The most likely sources of this disagreement lie in the methodology: (1) The discretized form of the equations relating CMB pressure to surface deformation deviates from the continuous form due to the use of large grid cells, and (2) The effects of using of staggered grid layouts, as opposed to a single inversion, are not clear.

Both of these drawbacks may be mitigated in future investigations by modeling the CMB pressure field continuously with a spherical harmonic expansion up to some moderate degree. Given the results of this study, future work that models the CMB pressure field with deformation data should explore multiple inversion parameters to check the robustness of the results. Additional insights could come from comparing the C_20_ predictions with more precise C_20_ observations, if such data becomes available.

This study is based on the assumption that axial angular momentum of the outer core is mediated by geostrophic flows, which is believed to be a reasonable assumption^24^. A more complicated model incorporating magnetic coupling^23^ needs further investigation, but is beyond the scope of this study. Another effect that may compromise GPS-observed surface deformation due to core-mantle pressure coupling is the correction of loading deformation due to atmospheric, oceanic, and hydrologic processes. The model we use for correction is from simulated models, not real measurements, and it is hard to estimate its error and uncertainty. We expect in the future when longer GPS time series and more reliable loading corrections are available, the surface deformation due to deep Earth processes can be more accurately observed by GPS.

The inverted outer core angular momentum using more recent (2004–2016) global GPS deformation data (Inversion B) indicates a reasonable agreement with the fitted LOD signal. However, we want to point out this study has not achieved a robust phase agreement and suffers from methodological limitations, precluding it from providing significant support for (or arguments against) the hypothesis of subdecadal core-mantle coupling. We have identified specific opportunities for methodological improvements and laid a foundation for their implementation, opening the door for the use of surface deformation data in Earth rotation studies.

## Electronic supplementary material

Supplementary Materials

Supplementary Figure S2: Flow Solution for Inversion A

Supplementary Figure S3: Flow Solution for Inversion B

## Data Availability

All source data used in this study can be found in the References and links provided in the text. Data products from this study are available from Zenodo at 10.5281/zenodo.1157059.
