# Supplementary material for: Earth’s Subdecadal Angular Momentum Balance from Deformation and Rotation Data
Source: Sci Rep. 2018 Sep 13;8:13761. doi: 10.1038/s41598-018-32043-8 (PMC6137097; doi:10.1038/s41598-018-32043-8)
Supplement: Supplementary file 1 — Supplementary Materials [file 41598_2018_32043_MOESM1_ESM.pdf]

Supplementary Materials for: Earth's Subdecadal Angular Momentum Balance from Deformation and Rotation Data

Andrew Watkins[1], **Yuning Fu**[1,*], **and Richard Gross**[2]

[1]Bowling Green State University, Geology Department, Bowling Green, OH, 43403, USA
[2]Jet Propulsion Laboratory, California Institute of Technology, Pasadena, CA, 91109, USA
[*]yfu@bgsu.edu

Supplementary Note on Figure 1

The selection of GPS stations used to display the result in Figure 1 is based on the same rules for selecting GPS stations later in the study: No more than 30% of missing data is allowed, and no gaps more than 365 days, during the time period being analyzed (1 January 2002−2014 in this case). There were 569 such GPS stations. Bilinear interpolation of the loading data was based on a mask file that incorrectly placed 46 of them in the ocean, and so those 46 did not have reliable loading data and were not used in this result.

The loading data consisted of non-tidal atmospheric loading (NTAL), non-tidal oceanic loading (NTOL), and hydrological loading (HYDL). The latter was provided at a daily temporal resolution, while the former two were provided at three-hour intervals. For NTAL and NTOL, the arithmetic mean was taken to create daily averages.

JPL's residual GPS time series have had a linear trend and seasonal estimate removed. In order to make the loading contributions comparable to the deformation, a linear trend was fit by least squares to the loading time series, using the detrend() function in MATLAB R2017, and removed. A seasonal estimate of the form $\lambda_1 \cos\left(\frac{2\pi}{365.25}t + \varphi_1\right) + \lambda_2 \cos\left(\frac{4\pi}{365.25}t + \varphi_2\right) + \varepsilon$ was then fit by nonlinear least squares to the loading time series and removed.

Supplementary Note on the Discrete Horizontal Gradient Operator

For each p_i we looped through all the adjacent pressure samples, and found the sample p_j that maximizes the scalar gradient: $(p_j - p_i)/\alpha_{ij}$. Consider a Cartesian coordinate system with Earth's center at its origin, and the z axis aligning with Earth's rotation axis. Let the Cartesian vectors $\mathbf{p1}$ and $\mathbf{p2}$ give the positions of the pressure sample points p_i and p_j, respectively. The first condition for the vector $\nabla_H p_i$ was that it lie on the plane tangent to the sphere at $\mathbf{p1}$. This is equivalent to forcing the dot product $\nabla_H p_i \cdot \mathbf{p1}$ to be zero. The second condition is that $\nabla_H p_i$ must point towards $\mathbf{p2}$. Consider the line intersecting both the origin and the point $\mathbf{p2}$. This line also intersects the plane tangent to the sphere at $\mathbf{p1}$ at another point $k\mathbf{p2}$, where $k > 0$ is some constant (whenever $\mathbf{p1} \cdot \mathbf{p2} > 0$). Thus, we required that $\nabla_H p_i = k\mathbf{p2} - \mathbf{p1}$. Combining these conditions gives:

$$\nabla_H p_i = k\mathbf{p2} - \mathbf{p1} = \frac{|\mathbf{p1}|^2}{\mathbf{p2} \cdot \mathbf{p1}}\mathbf{p2} - \mathbf{p1}$$

The vector $\nabla_H p_i$ was then rescaled to the magnitude of the scalar gradient $(p_j - p_i)/\alpha_{ij}$, and the flow vectors **u** were computed directly from equation (6) in the main text.

Supplementary Note on the Moments of Inertia of Cylindrical Annuli of Outer Core Fluid

Consider the region of outer core fluid lying outside a single cylinder intersecting the CMB at latitudes $\pm\psi_o$. Assuming constant density, the axial moment of inertia of this fluid is:

$$I_z = \iiint_Q (x^2 + y^2) dm = \iiint_Q (x^2 + y^2)\rho_{OC} dV = \rho_{OC} \iiint_Q (x^2 + y^2)\, dx\, dy\, dz$$

where Q describes the geometry of the annulus. The transformation to spherical coordinates with radius r, latitude, and longitude ϕ was then used: $x = r\cos\psi\cos\phi$, $y = r\cos\psi\sin\phi$, and $z = r\sin\psi$. Multiplying the integrand by $r^2\cos\psi$, the Jacobian determinant of this transformation, completes the change of variables:

$$I_z(\psi_o) = \rho_{OC} \int_0^{2\pi} \int_{-\psi_o}^{+\psi_o} \int_{r_{CMB}\frac{\cos\psi_o}{\cos\psi}}^{r_{CMB}} r^4 \cos^3\psi\, dr\, d\psi\, d\phi$$

We then have:

$$I_z(\psi_o) = \frac{4\pi\rho_{OC} r_{CMB}^5}{5} \sin\psi_o \left(1 - \frac{\sin^2\psi_o}{3} - \cos^4\psi_o\right)$$

Supplementary Note on the Computation of the C_{20} Coefficient of the Gravitational Field

At Earth's surface, the zonal part of the gravitational potential may be expressed as[1]:

$$U(r_E, \Omega) = \frac{GM_E}{r_E} \sum_{l=0}^{\infty} C_{l0} P_{l0}(\cos\theta)\sqrt{2l+1}$$

where Ω is an abbreviation for the angles θ and ϕ, the C_{lm} are the unitless Stokes' coefficients, and the P_{l0} are the associated Legendre functions of degree l and order 0 given by:

$$P_{l0}(\mu) = \frac{1}{2^l l!} \frac{d^l}{d\mu^l} (\mu^2 + 1)^l$$

We converted the ΔU to C_{20} first by considering the spherical harmonic functions[1] Y_{lm}

$$Y_{lm}(\Omega) = (-1)^m P_{lm}(\cos\theta) e^{im\phi} \sqrt{\frac{(2l+1)(l-m)!}{4\pi(l+m)!}}$$

Using the previous three equations, the surface gravitational potential can be rewritten as:

$$U(r_E, \Omega) = \frac{2GM_E\sqrt{\pi}}{r_E}\sum_{l=0}^{\infty} C_{l0} Y_{l0}(\Omega)$$

Multiplying by the complex conjugate Y_{20}^* of the degree-2 spherical harmonic Y_{20}, and integrating with respect to Ω gives:

$$\int_Q U(r_E, \Omega) Y_{20}^*(\Omega) d\Omega = \frac{2GM_E\sqrt{\pi}}{r_E}\sum_{l=0}^{\infty} C_{l0} \int_Q Y_{l0}(\Omega) Y_{20}^* d\Omega$$

where Q gives all possible values of Ω on a sphere and $d\Omega = \sin\theta\, d\theta\, d\phi$. The summation on the right-hand side of the preceding equation is equal to C_{20} due to the normalization of the spherical harmonics[1]. Evaluating Y_{20}^* gives:

$$C_{20} = \frac{r_E\sqrt{5}}{8GM_E\pi}\int_Q U(r_E, \theta, \phi)(3\cos^2\theta - 1)\sin\theta\, d\theta d\phi$$

The gravitational potential U was given for a finite number of grids on Earth's surface, so the integration was considered as the sum of the integrations over each of these grids, and U was considered constant within the grids:

$$C_{20} = \frac{r_E\sqrt{5}}{8GM_E\pi}\sum_i U_i \int_{\phi_{i1}}^{\phi_{i2}} \int_{\theta_{i1}}^{\theta_{i2}} (3\cos^2\theta - 1)\sin\theta\, d\theta\, d\phi$$

$$C_{20} = \frac{r_E\sqrt{5}}{8GM_E\pi}\sum_i U_i(\phi_{i1} - \phi_{i2})(\cos^3\theta_{i2} - \cos\theta_{i2} - \cos^3\theta_{i1} + \cos\theta_{i1})$$

where the ϕ_{ij} and θ_{ij} are the appropriate boundaries for the ith grid cell.

Supplementary Table S1: The locations of grid cell centers for all six inversions.

Boundary latitude and longitude lines for the grids are placed halfway in between adjacent grid cells. Data below is in the following format:
Inversion #, Grid cell #, Grid cell center longitude (radians), Grid cell center latitude (radians)

1.000000e+00 1.000000e+00 -2.967060e+00 1.047198e+00
1.000000e+00 2.000000e+00 -8.726646e-01 1.047198e+00
1.000000e+00 3.000000e+00 1.221730e+00 1.047198e+00
1.000000e+00 4.000000e+00 -2.967060e+00 0.000000e+00
1.000000e+00 5.000000e+00 -8.726646e-01 0.000000e+00
1.000000e+00 6.000000e+00 1.221730e+00 0.000000e+00

1.000000e+00 7.000000e+00 -2.967060e+00 -1.047198e+00
1.000000e+00 8.000000e+00 -8.726646e-01 -1.047198e+00
1.000000e+00 9.000000e+00 1.221730e+00 -1.047198e+00
2.000000e+00 1.000000e+00 0.000000e+00 1.570796e+00
2.000000e+00 2.000000e+00 -2.967060e+00 5.235988e-01
2.000000e+00 3.000000e+00 -8.726646e-01 5.235988e-01
2.000000e+00 4.000000e+00 1.221730e+00 5.235988e-01
2.000000e+00 5.000000e+00 -2.967060e+00 -5.235988e-01
2.000000e+00 6.000000e+00 -8.726646e-01 -5.235988e-01
2.000000e+00 7.000000e+00 1.221730e+00 -5.235988e-01
2.000000e+00 8.000000e+00 0.000000e+00 -1.570796e+00
3.000000e+00 1.000000e+00 -2.268928e+00 1.047198e+00
3.000000e+00 2.000000e+00 -1.745329e-01 1.047198e+00
3.000000e+00 3.000000e+00 1.919862e+00 1.047198e+00
3.000000e+00 4.000000e+00 -2.268928e+00 0.000000e+00
3.000000e+00 5.000000e+00 -1.745329e-01 0.000000e+00
3.000000e+00 6.000000e+00 1.919862e+00 0.000000e+00
3.000000e+00 7.000000e+00 -2.268928e+00 -1.047198e+00
3.000000e+00 8.000000e+00 -1.745329e-01 -1.047198e+00
3.000000e+00 9.000000e+00 1.919862e+00 -1.047198e+00
4.000000e+00 1.000000e+00 0.000000e+00 1.570796e+00
4.000000e+00 2.000000e+00 -2.268928e+00 5.235988e-01
4.000000e+00 3.000000e+00 -1.745329e-01 5.235988e-01
4.000000e+00 4.000000e+00 1.919862e+00 5.235988e-01
4.000000e+00 5.000000e+00 -2.268928e+00 -5.235988e-01
4.000000e+00 6.000000e+00 -1.745329e-01 -5.235988e-01
4.000000e+00 7.000000e+00 1.919862e+00 -5.235988e-01
4.000000e+00 8.000000e+00 0.000000e+00 -1.570796e+00
5.000000e+00 1.000000e+00 -1.570796e+00 1.047198e+00
5.000000e+00 2.000000e+00 5.235988e-01 1.047198e+00
5.000000e+00 3.000000e+00 2.617994e+00 1.047198e+00
5.000000e+00 4.000000e+00 -1.570796e+00 0.000000e+00
5.000000e+00 5.000000e+00 5.235988e-01 0.000000e+00
5.000000e+00 6.000000e+00 2.617994e+00 0.000000e+00
5.000000e+00 7.000000e+00 -1.570796e+00 -1.047198e+00
5.000000e+00 8.000000e+00 5.235988e-01 -1.047198e+00
5.000000e+00 9.000000e+00 2.617994e+00 -1.047198e+00
6.000000e+00 1.000000e+00 0.000000e+00 1.570796e+00
6.000000e+00 2.000000e+00 -1.570796e+00 5.235988e-01
6.000000e+00 3.000000e+00 5.235988e-01 5.235988e-01
6.000000e+00 4.000000e+00 2.617994e+00 5.235988e-01
6.000000e+00 5.000000e+00 -1.570796e+00 -5.235988e-01
6.000000e+00 6.000000e+00 5.235988e-01 -5.235988e-01
6.000000e+00 7.000000e+00 2.617994e+00 -5.235988e-01
6.000000e+00 8.000000e+00 0.000000e+00 -1.570796e+00

Supplementary Table S2: Values for Physical Parameters.

Parameter and source	Value
ρ_{OC} (outer core's mean density)[2]	$1.12393 \times 10^4 \frac{kg}{m^3}$
$\bar{\rho}_E$ (mean density of Earth)[3]	$5.5 \times 10^3 \frac{kg}{m^3}$
g (gravitational acceleration at Earth's surface)[3]	$9.81 \frac{m}{s^2}$
h_n , $n = 1, \ldots, 10$ (load Love numbers for a pressure load from the core acting on the surface)[4]	-1.42523, 0.65898, 0.35221, 0.19433, 0.11313, 0.06797, 0.04135, 0.02519, 0.01530, 0.00924 (respectively)
k_n , $n = 1, \ldots, 10$ (load Love numbers for a pressure load from the core acting on the surface)[4]	0.22776, 0.31735, 0.10924, 0.04381, 0.01990, 0.00976, 0.00500, 0.00263, 0.00140, 0.00075 (respectively)
r_{ICB} (radius of the inner core)[2]	1.2215×10^6 m
r_{CMB} (radius of the outer core[2]	3.48×10^6 m
r_E (radius of Earth)[2]	6.731×10^6 m
$\bar{\omega}_E$ (Earth's mean rotation rate)[5]	$7.292115 \times 10^{-5} \frac{rad}{s^2}$
M_E (mass of Earth)[2]	5.974×10^{24} kg
G (universal gravitational constant)[5]	$6.67259 \times 10^{-11} \frac{m^3}{s^2 kg}$
C_{mc} (axial moment of inertia of the mantle and crust)[6]	7.1236×10^{37} kg m^2

Supplementary Figure S1: LOD Time Series.

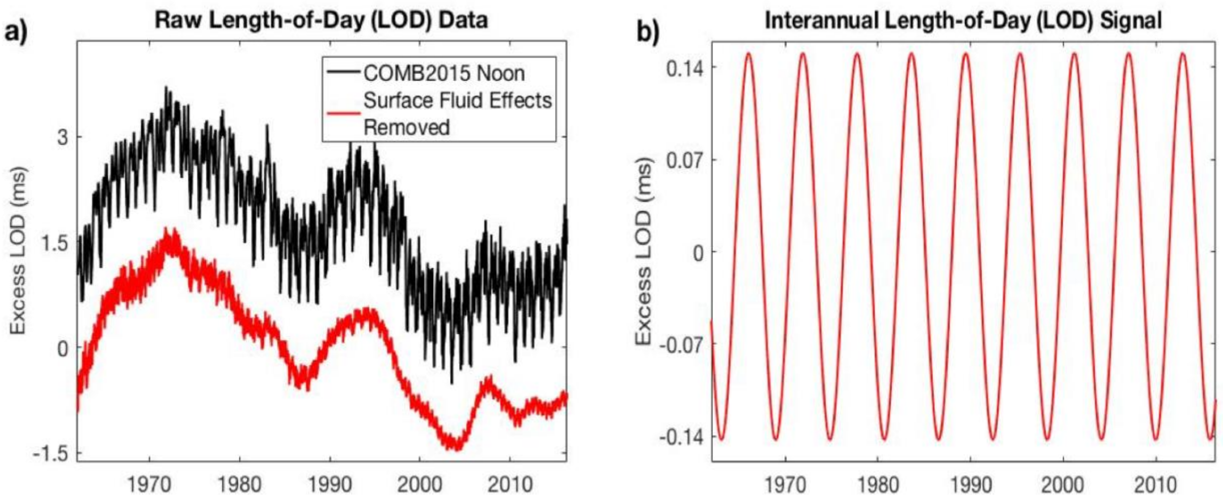

Figure S1. a) Removal of atmospheric and oceanic effects reduces the seasonal variability in the COMB2015 Noon Length-of-Day (LOD) time series. b) The isolated LOD signal has a period of 5.85 years and an amplitude of 0.15 ms.

Supplementary Figure S2: Flow Solution for Inversion A

Figure S2. Included in a separate file. The evolution over time of the flow solutions for inversion A. The projection of the continents' outlines onto the core-mantle boundary has been included for reference.

Supplementary Figure S3: Flow Solution for Inversion B

Figure S3. Included in a separate file. The evolution over time of the flow solutions for inversion B. The projection of the continents' outlines onto the core-mantle boundary has been included for reference.

References for Supplementary Material

1. Chao, B., & Gross, R. Changes in the Earth's rotation and low-degree gravitational field induced by earthquakes. *Geophy. J. R. Astron. Soc.* **91,** 569–596. https://doi.org/10.1111/j.1365-246X.1987.tb01659.x (1987).

2. Dziewonski, A., & Anderson, D. Preliminary reference Earth model. *Phys. Earth Planet. Int.* **25,** 297–356. https://doi.org/10.1016/0031-9201(81)90046-7 (1981).

3. Mussett, A., & Khan, M. *Looking Into the Earth: An Introduction to Geological Geophysics*. 1st ed. Cambridge: Cambridge University Press (2000).

4. Fang, M., Hager, B., & Kuang, W. Degree one loading by pressure variations at the

CMB. *Journal of Earth Science*. **24(5),** 736–749. https://doi.org/10.1007/s12583-013-0367-5 (2013).

5. Groten, E. Fundamental parameters and current (2004) best estimates of the parameters of common relevance to astronomy, geodesy, and geodynamics. *J. Geodesy*. **77,** 724–731. https://doi.org/10.1007/s00190-003-0373-y (2004).

6. Mathews, P., Buffett, B., Herring, T., & Shapiro II. Forced nutations of the Earth: Influence of inner core dynamics, 2. Numerical results and comparisons. *J. Geophys. Res.* **96,** 8243–8257. https://doi.org/10.1029/90JB01956 (1991).
