# Supplementary figures and images for: Earth’s Subdecadal Angular Momentum Balance from Deformation and Rotation Data
Source: Sci Rep. 2018 Sep 13;8:13761. doi: 10.1038/s41598-018-32043-8 (PMC6137097; doi:10.1038/s41598-018-32043-8)

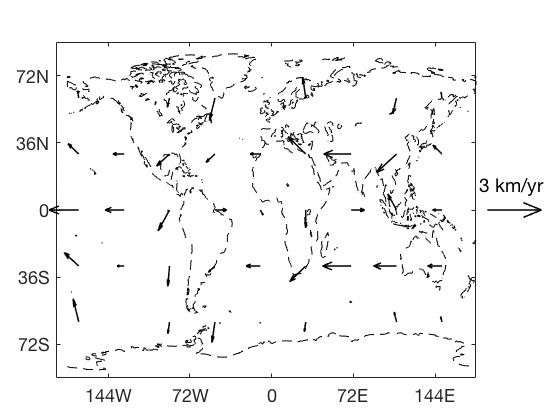

Supplement: Supplementary file 2 — Supplementary Figure S2: Flow Solution for Inversion A [file 41598_2018_32043_MOESM2_ESM.gif]

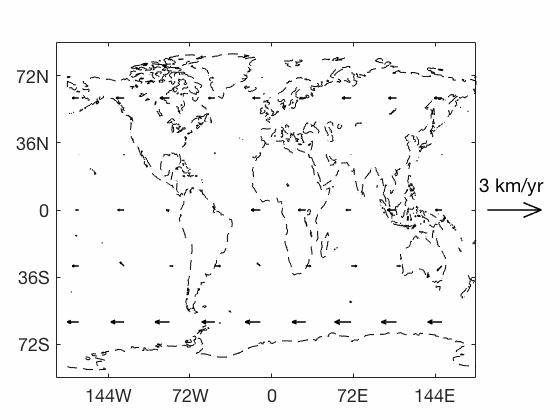

Supplement: Supplementary file 3 — Supplementary Figure S3: Flow Solution for Inversion B [file 41598_2018_32043_MOESM3_ESM.gif]
